# Low prevalence of hepatitis delta infection in Cuban HBsAg carriers: Prospect for elimination

**DOI:** 10.3389/fmed.2022.1069372

**Published:** 2023-02-01

**Authors:** Licel de los Ángeles Rodríguez Lay, Zexi Tan, Maria Caridad Montalvo Villalba, Marcia Samada Suárez, Marité Bello Corredor, Dayesi López Hernández, Barbara Marrero Sánchez, Lidunka Valdés Alonso, Aurélie Sausy, Judith M. Hübschen

**Affiliations:** ^1^National Reference Laboratory of Viral Hepatitis, Department of Virology, Institute of Tropical Medicine “Pedro Kourí”, Havana, Cuba; ^2^Centro de Investigaciones Médico Quirúrgicas, Havana, Cuba; ^3^Clinical and Applied Virology Group, Department of Infection and Immunity, Luxembourg Institute of Health, Esch-sur-Alzette, Luxembourg

**Keywords:** hepatitis delta virus (HDV), hepatitis B virus, HBsAg carriers, hepatitis B vaccination, HDV elimination

## Abstract

**Introduction:**

Infection with hepatitis delta virus (HDV) is one of the most severe hepatitis B virus (HBV) complications, with a more rapid progression to cirrhosis and an increased risk of hepatic decompensation and death. Data on HDV infection in Cuba are limited. The aims of our study were to determine the HDV prevalence in HBsAg carriers and to characterize the HDV strains circulating. The data were used to assess the possibility of HDV elimination in the Cuban HBV epidemiological setting.

**Methods:**

Five hundred and two serum samples from the same number of HBsAg carriers collected in the period 2006–2019 from all over the country were tested for anti-HDV total antibodies. If positive, the samples were analyzed for HDV-RNA using Real-Time RT-PCR targeting the ribozyme and HD antigen domains followed by genotyping based on phylogenetic analysis.

**Results:**

Two samples were anti-HDV positive [0.39% (95% CI 0.11–1.44)]. One of them was also HDV-RNA positive. Clinically, the patient with active HDV infection had compensated liver cirrhosis. Phylogenetic analysis showed that the virus belonged to genotype 1 and thus clustered with contemporary strains from North America, Europe, Middle East, and Asia.

**Discussion:**

This is the first HDV study, including molecular detection and virus characterization, done after the introduction of the universal childhood anti-hepatitis B vaccination. The very low prevalence of HDV infection in HBsAg carriers combined with the high HBV vaccination coverage of all newborn children, of previously identified risk groups, and of the general population currently under 40 years of age suggests that HDV elimination is feasible in Cuba if the success in HBV control is maintained.

## Introduction

Hepatitis delta virus (HDV) is a unique RNA virus requiring hepatitis B virus (HBV) for replication and infection of hepatocytes (1). Recent data estimated that more than 10% of people with chronic HBV infection are co-infected with HDV, yielding a global prevalence of 0.80% in the general population, and resulting in a total of 48–60 million persons presumably infected with HDV worldwide (2, 3).

The prevalence of HDV is usually assessed based on HDV antibody positivity among HBsAg carriers. Implementation of routine hepatitis B vaccination of children and other population groups in industrialized countries resulted in a considerable decrease of HDV prevalence (4). The impact of the disease in low-income countries is largely unknown due to a lack of awareness and of adequate diagnostic tools. In addition, available treatments are often suboptimal.

Hepatitis delta virus belongs to the genus *Deltavirus* and was recently reclassified into a new family Kolmioviridae and a new realm called *Ribozyviria* (5). HDV has a genome size of 1.7 kilobases (kb) and phylogenetic analyses have distinguished eight genotypes 1–8, which differ by up to 30% in their RNA sequence. Genotype 1 is present worldwide, while genotypes 2–8 were found in more specific geographical areas: HDV-2 and –4 are of Asian origin. HDV-3 is found in South America and HDV-5 to 8 were reported mainly from Africa and more recently in Brazil (1, 6–8).

Hepatitis delta virus genotype 1 has been associated with a broad spectrum of pathogenicity, while HDV genotype 2 is normally linked to milder forms of liver disease. HDV-3 has been reported in connection with a severe form of fulminant hepatitis, while HDV-4 is often related to mild liver disease although a variant of genotype 4 seems to increase the risk for progression to chronic hepatitis and cirrhosis. The more recently identified genotypes 5–8 from Africa are less well characterized (6–8).

Cuba is an HBV low prevalence country with a predominance of subgenotype A2 and HBsAg serotype adw2 (9). Three doses of hepatitis B vaccine are recommended at 2, 4, and 6 months of age and 1 dose for newborns within 24 h of birth. The high vaccination coverage of more than 95% in the study period has led to HBV control, with an incidence rate of 0.5/100.000 population in 2020 (10). In line with the WHO global strategy of hepatitis elimination by 2030, Cuba has set up a National Strategic Plan for the Prevention and Control of Sexually Transmitted Diseases including viral hepatitis (11). However, data regarding HDV infection is limited with only one serological study done 32 years ago, when the epidemiological context of HBV infection was very different from today (12). Therefore, the main aims of our study were to determine the HDV prevalence in Cuban HBsAg carriers and to characterize the HDV strains present in the country. The data were used to assess the possibility of HDV elimination in the Cuban HBV epidemiological setting. Additionally, the study provides reliable information for worldwide HDV prevalence estimates to guide international HBV and HDV control programs toward global elimination.

## Materials and methods

### Study population

**FIGURE 1:**
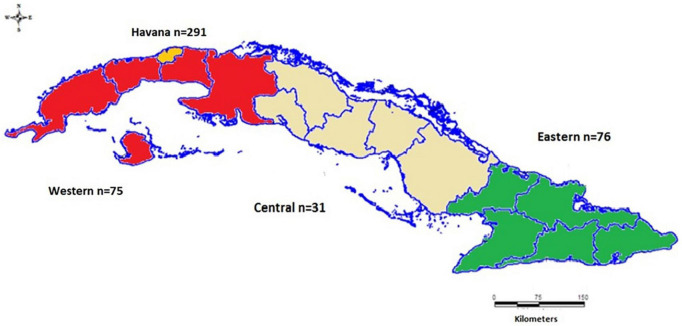
Map showing the main regions of Cuba and Havana as well as the number of samples originating from there.

Five hundred and two serum samples received at the National Reference Laboratory of Viral Hepatitis at the Institute of Tropical Medicine Pedro Kouri (IPK) to confirm HBsAg positivity and/or to perform HBV molecular diagnosis between 2006 and 2019 from all over the country were included in this study. The sera had been stored at −20°C and sample information was retrieved from laboratory books or dedicated databases. Two samples had been collected in 2006, 2 in 2007, 9 in 2008, 6 in 2009, 1 in 2011, 37 in 2014, 100 in 2015, 92 in 2016, 143 in 2017, 86 in 2018, and 24 in 2019. Two hundred and ninety-one samples were received from hospitals in Havana (Tertiary Care), 76 sera were from the Eastern, 31 from the Central and 75 from the Western part of Cuba (Figure 1). Twenty-nine samples lacked data about the geographical origin of the patients. In the context of our study, the doctor in charge of a patient with detectable HDV-RNA was informed and a new serum sample for virological follow-up was requested.

Some demographic, epidemiological, clinical, and virological characteristics of the study population are shown in Table 1.

**TABLE 1:** Demographic, epidemiological, clinical, and virological characteristics of the 502 study participants, Cuba 2006–2019.

Parameters		*N*	%
Age	Children (0–≤18 years)	24	4.78
	Adults (>18 years), median age 42 years, and interquartile range [33–51]	188	37.45
	No data (adults)	290	57.77
Sex	Male	63	12.54
	Female	112	22.31
	No data	327	65.13
Purpose of HBV testing	Follow up of children of HBsAg + mothers	19	3.78
	Surveillance of pregnant women	91	18.12
	Surveillance of acute infection	6	1.19
	Diagnosis of severe hepatitis	7	1.39
	Follow up of chronic HBV infection	220	43.82
	Others	159	31.6
Co-infections	HBV/HCV	1	0.19
	HBV/HIV	90	17.92
	HBV/HCV/HIV	3	0.59
	None known	408	81.2
HBV-DNA (PCR or qPCR)	Detectable HBV DNA	146	29.08
	Non-detectable HBV DNA	123	24.5
	Not tested	233	46.4

### Ethics approval and consent to participate

The study was conducted in compliance with the Declaration of Helsinki and using Good Laboratory Practices. The specimens tested for this research were residual samples received for HBV serological and/or molecular analysis. The research was approved by the Ethics Committee of the Institute for Tropical Medicine in Havana, Cuba (CEI-IPK 05-16). In case of positive results, the doctor in charge was informed. Written informed consent was obtained from the patient with active HDV infection (HDV-RNA positive) for reviewing the clinical history and for taking serum samples for the follow-up of the HBV and HDV infection status.

### Laboratory investigations

Detection and confirmation of the HBsAg was done with reagents and technology from Tecnosuma Internacional S.A. (UMELISA HBsAg PLUS and UMELISA HBsAg Confirmatory test). Total antibodies against HDV were detected with commercial enzyme linked immunosorbent assays (Dia.Pro, Italy). Both assays were performed according to the manufacturer’s instructions.

Viral RNA was extracted from 140 μL of serum using the QIAamp Viral RNA mini kit (Qiagen GmbH, Hilden, Germany). Five μL of RNA were denatured with 45 ng random primers and 10 nmol nucleotides for 5 min at 72°C. Reverse transcription (RT) was then performed for 80 min at 50°C using 200 U SuperScript III reverse transcriptase and 40 U RNaseOUT recombinant RNase inhibitor (Invitrogen, Karlsruhe, Germany) (13). RT-PCR for HDV detection was done as described previously (14). Five μL of cDNA were added to TaqMan Universal PCR Master Mix (Applied Biosystems, Foster City, CA, United States) as well as 0.5 μM primers and 2.5 μM probe.

Fragments for HDV genotyping were amplified using primers 480as, 710s, 1302das, rv900, fw900_2 and 320ds (13) as well as 1170s (5′-ctcgtcttchhcggtcaacctc-3′, Andernach et al., unpublished). cDNA synthetized as previously described was used with the Phusion High-Fidelity DNA Polymerase (New England Biolabs, Ipswich, United States). The PCR was done using 0.8 μM of primers, 0.5 mM of MgCl2 and 0.02 U/μl of the polymerase and the following amplification conditions: 98°C for 30 s, 40× (98°C for 10 s, 54°C for 30 s, 72°C for 60 s) and a final elongation at 72°C for 7 min. In addition, RNA was used with the One step RT-PCR kit (Qiagen GmbH, Hilden, Germany) and 0.5 μM of primers, 1.5 mM of MgCl2 and 1 μl of enzyme mixture per reaction. The cycling conditions were: 50°C for 30 min, 95°C for 15 min, 40× (95°C for 30 s, 54°C for 30 s, 72°C for 60 s) and a final elongation step at 72°C for 10 min.

PCR products were purified using the QIAquick gel extraction kit (Qiagen GmbH, Hilden, Germany) and sequenced using the ABI Prism Big Dye Terminator cycle sequencing reaction kit (Applied Biosystems, Foster City, CA, United States) in an ABI3130xl genetic analyzer.

Sequences were edited with SeqScape v2.5 software (Applied Biosystems) and BioEdit Sequence Alignment Editor (version 7.0.9.0, Ibis Biosciences, Carlsbad, CA, United States) and aligned with reference sequences of the 8 HDV genotypes and with similar sequences obtained by BLAST.^1^ Phylogenetic analyses were conducted with MEGA version 6^2^ and phylogenetic trees were constructed using the neighbor-joining method and the Kimura 2-parameter model. The bootstrap method with 1,000 replications was used as measure of the robustness of each node.

Sequences obtained during this study were submitted to the GenBank Nucleotide Sequence Database under accession number: [MW273290].

### Statistical analyses

A Microsoft Excel 2010 database was created for data analysis. The Chi-square test and the confidence interval (CI) calculations were done using GraphPad 7.0. Results were considered to be statistically significant when *p* < 0.05.

## Results

Two of the 502 sera were positive for HDV antibodies (0.39%, [95% CI 0.11–1.44]). One of the positive sera had been collected in 2015, the other in 2017. Both positive samples were from male patients, between 50 and 60 years old and with chronic HBV infection. None of the two patients had an HCV or HIV co-infection. Also, in both patients HBeAg was negative and HBV-DNA was undetectable. HDV-RNA was detected only in one of them.

Clinically, this patient had compensated liver cirrhosis with portal hypertension with only splenomegaly and esophageal varices grade 1. Serum alanine transaminase (ALT), aspartate transaminase (AST), and gamma-glutamyl transferase (GGT) levels were increased, while the alkaline phosphatase (ALP) and total serum bilirubin (TSB) values were in the normal range. Other hematological and biochemical parameters like creatine, albumin, platelets, and International Normalized Ratio (INR) were also in the normal range. The patient had been under Lamivudine therapy during the last 5 years. After the HDV diagnosis, he received Pegylated Interferon Alpha therapy during 1 year. At the end of the treatment the HDV-RNA and the HBV-DNA were undetectable, while the HBsAg remained positive and the enzymes ALT and AST were in the normal range [median ALT before treatment 117 vs. median post treatment 49 (*p* = 0.05), AST: 89 vs. 51 (*p* > 0.05)]. The GGT level remained high as a marker of fibrosis.

**FIGURE 2:**
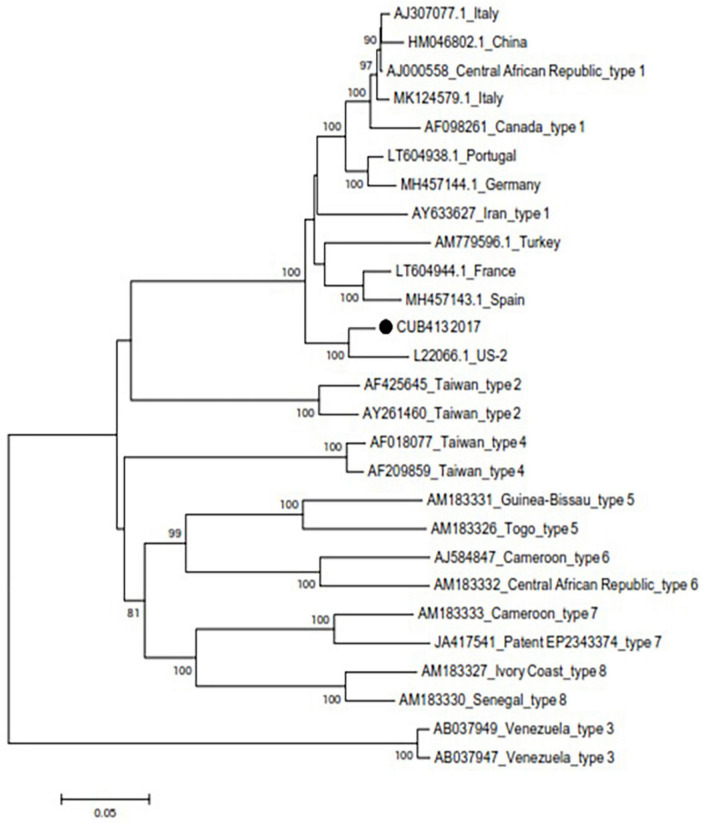
Phylogenetic tree based on the neighbor-joining method and the Kimura 2-parameter model using the partial genome of hepatitis delta virus (HDV). The sequence from Cuba is marked with a black dot. Each reference sequence is identified by GenBank accession number, country of origin, and genotype. Other genotype 1 sequences obtained by BLAST are identified by GenBank accession number and country of origin. Bootstrap values above 70 are shown at the nodes and genetic distance may be estimated based on the bar below the tree.

Sequence data covering nucleotide positions 1–439 and 660–1,682 of the HDV genome (according to US-2 sequence, accession number L22066.1) were obtained. Phylogenetic analysis of the combined 1,462 nucleotides showed that the sequence from Cuba belonged to genotype 1 and thus clustered with contemporary strains from North America (United States), Europe (Spain, Portugal, France, Germany, and Italy), Middle East (Iran and Turkey), and Asia (China) (Figure 2).

## Discussion

Although overlapping, the prevalence of HDV does not always coincide with that of HBV. In the past years, HBV vaccination and sexual limitations driven by the risk of AIDS have led to the control of HBV with a significant reduction of the number of HBsAg carriers in many countries. Deprived of HBV infections, the circulation of HDV has noticeably declined principally in the industrialized world (4, 15).

The anti-HDV prevalence found in the present study is very low. In the only other study done in Cuba in 1988, an anti-HDV positivity of 8.3% was detected (11). While we cannot exclude false positive results in the previous study, the ELISA kit used in the present study is expected to reliably detect HDV antibodies with reported sensitivity and specificity values of above 98% (16). The difference may be related to the success of the Cuban hepatitis B prevention and control program, since HBV vaccination is the most influential factor concerning the prevalence of both diseases (17). The vaccination strategy included in the National Immunization Program in 1992 comprised vaccination of all newborn children with the first dose provided in maternity hospitals, as well as vaccination of risk groups to prevent infection before potential exposure. In 2000, a vast vaccination campaign targeting all people less than 20 years of age was done (18). Therefore, the majority of the Cuban population currently under 40 years old is vaccinated against hepatitis B. After 28 years of nationwide vaccination, the rate of new infections has been drastically reduced (from 20.3/100.000 population in 1992 over 2.2/100.000 in 2001 to 0.5/100.000 in 2020) (10). Other measures for HBV control included the screening of all pregnant women, of blood and blood products, the surveillance of children born to HBsAg positive mothers using serological or more recently molecular techniques and education of the population. The considerable reduction of HBV cases may have influenced the HDV epidemiology and thus its currently very low prevalence rate.

Consistent global data on the prevalence of HDV are lacking because of different reasons such as lack of testing of HBsAg carriers for HDV infection or non-availability of high quality anti-HDV antibody assays and there are considerable geographical variations (3, 19, 20). Countries in Asia have reported prevalence rates between 4.4 and 60% (21–23), while in sub-Saharan Africa HDV prevalence ranged from 0 to 50% in relation to the clinical picture of the patients (12). In Europe, the situation is complex because of immigration (24–27) and also countries in the Western world with low HBV prevalence, such as the United States, report diverse HDV prevalence rates (28, 29). The Western Amazonia represents one of the places with the highest prevalence of HDV in the world (30), while other Latin American and Caribbean countries have low prevalence rates (31, 32). HDV control or elimination, however, is only possible via successful HBV immunization programs (17).

The HDV RNA positive patient was born before the introduction of routine childhood HBV vaccination in Cuba. In a recent study analyzing the characteristics of HDV patients from different regions worldwide, the authors stated that men are more frequently infected with HDV than women and that regional differences concerning disease epidemiology and management exist (33).

The interaction between HBV and HDV is complex and multiple virological and host-related factors may be involved. HDV may be temporarily or permanently the dominant virus (34) and HBV DNA levels are often low or even undetectable in patients with chronic HDV infection (33, 35). Also, in the present study both anti-HDV positive cases were HBeAg negative and HBV DNA was undetectable, although the HBV genotype may play a role in the course of chronic hepatitis D. For instance, HBV genotype C has been associated with adverse outcomes (cirrhosis, hepatocellular carcinoma, or mortality) in patients with chronic hepatitis D (36). In Cuba, HBV genotypes A and D are prevalent (9). Since these two genotypes seem to have a very different influence on HDV infectivity (37), it would be interesting to know with which HBV genotype HDV positive patients are infected to anticipate disease outcome.

For the HDV-PCR positive patient, RNA was undetectable after 1 year of Interferon treatment and the liver enzymes were normal suggesting a virological and biochemical response. Although this is the first choice of HDV treatment and decreases the HDV viral load in most patients, only 25% of the patients have undetectable levels of RNA afterward (1, 6, 38, 39). A follow up is needed to verify whether the treatment has a sustained virological response over time, even beyond 24 weeks after treatment suspension (1). Most patients with HBV/HDV coinfection have high levels of ALT, AST and TSB and maintain a stable condition for a long time before decompensation or hepatic carcinoma occur (8, 33, 40). Disease progression may be influenced by the HDV genotype, with types 1 and 3 being linked to a more severe disease than genotypes 2 and 4 (6, 35, 40).

## Conclusion

This is the first HDV study, including molecular detection and virus characterization, done after the introduction of the universal childhood anti-hepatitis B vaccination. The very low prevalence of HDV infection in HBsAg carriers combined with the high HBV vaccination coverage of all newborn children, of previously identified risk groups, and of the general population currently under 40 years of age, suggest that HDV elimination is feasible in Cuba if the success in HBV control is maintained.

## Data availability statement

The data presented in this study are deposited in the Genbank repository (https://www.ncbi.nlm.nih.gov/genbank/), accession number: MW273290.

## Ethics statement

The study was conducted in compliance with the Declaration of Helsinki and using Good Laboratory Practices. The specimens tested for this research were residual samples received for HBV serological and/or molecular analysis. The research was approved by the Ethics Committee of the Institute for Tropical Medicine in Havana, Cuba (CEI-IPK 05-16). In case of positive results, the doctor in charge was informed. Written informed consent was obtained from the patient with active HDV infection (HDV-RNA positive) for reviewing the clinical history and for taking serum samples for the follow-up of the HBV and HDV infection status.

## Author contributions

LÁ and JH: conceptualization, resources, supervision, and project administration. LÁ, MV, ZT, MC, DH, BS, MS, LA, and AS: methodology. LÁ, AS, and JH: software. LÁ, MV, MS, and AS: validation. LÁ, MV, MS, ZT, MC, DH, BS, AS, and JH: analysis. LÁ, MS, and ZT: investigation. LÁ, MS, and LA: data curation. LÁ: writing—original draft preparation. LÁ, MS, and JH: writing—review and editing. LÁ, MS, AS, and JH: visualization. JH: funding acquisition. All authors read and agreed to the published version of the manuscript.

## Footnotes

1
https://blast.ncbi.nlm.nih.gov

2
www.megasoftware.net
